# Transverse Distraction of Great Toe to Enlarge the Donor Site before Finger Reconstruction

**DOI:** 10.1111/os.14229

**Published:** 2024-09-05

**Authors:** Liwen Hao, Kai Rong, Chao Chen, Zhidian Hou, Yaxing Wang, Xu Tian, Zengtao Wang

**Affiliations:** ^1^ Shandong Provincial Hospital Shandong University Jinan China; ^2^ Department of Hand & Foot Surgery and Reconstructive Microsurgery Shandong Provincial Hospital Affiliated to Shandong First Medical University Jinan China; ^3^ Department of Hand and Foot Surgery Fourth People's Hospital of Jinan Jinan China; ^4^ Shandong First Medical University Jinan China

**Keywords:** Donor‐site Morbidity, Distraction Osteogenesis, Ilizarov Technique, Partial Great Toe Flap, Toe‐to‐hand Transfer

## Abstract

Partial great toe transfer is widely used in finger reconstruction. Although satisfactory results have been reported at the recipient's hand, the donor foot still presents with many problems due to the large amount of tissues harvested. In this study, the Ilizarov technique was utilized to enlarge the great toe in order to minimize the amount of tissue sacrificed of the donor foot. In this retrospective study, 23 patients (30 toes) underwent transverse distraction of the great toe for finger reconstruction from September 2020 to December 2022. The width of the contralateral normal finger was set as the objective width gained of distraction. At the last follow‐up, the changes of bone, toenail, plantar skin, vessel, and nerve of the great toe were measured, and postoperative complications were assessed. The time for active distraction was 46.1 ± 8.3 days, with a widening rate of 0.41 ± 0.08 mm/day. Counting in the time for latency and consolidation, the time of treatment with external fixation was 84 ± 11.9 days. At the last follow‐up, the average width of the distal phalanx of the great toe increased from 13.1 to 28.1 mm (*p* < 0.001). The width of the toenail increased from 15.8 to 30.3 mm (*p* < 0.001), and the width of the plantar pulp increased from 25.6 to 38.8 mm (*p* < 0.001). Computed tomography angiography (CTA) and Doppler ultrasound confirmed that the digital arteries and nerves of the great toe were intact after distraction surgery. Two patients needed revision surgery due to complications of pin loosening or premature consolidation. With the help of the Ilizarov technique, the great toe is effectively enlarged after transverse distraction. Multiple tissues of the great toe, including bone, nail, and plantar skin, are regenerated, and more tissues were preserved after toe‐to‐hand transfer. To the best of our knowledge, this is a novel method to enlarge the donor site for finger reconstruction.

## Graphical abstract

Ilizarov technique is used to enlarge the great toe through transverse distraction. An L‐shaped incision was made on the dorsal aspect of distal great toe. Then four Kirschner pins were inserted on the distal phalanx and osteotomy was performed. After slow distraction by two mini external fixation frames, the donor's great toe was enlarged for finger reconstruction.

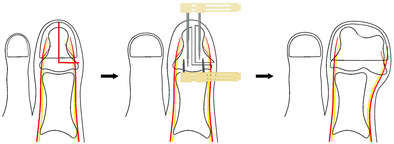

## Introduction

In the past decades, partial great toe transfer has become increasingly popular in surgical finger reconstruction.^1^, ^2^, ^3^, ^4^ Multiple tissues, including bone, skin, and nail, are harvested from the great toe to repair finger defects. As a “like‐to‐like” reconstruction, satisfactory functional and esthetic outcomes have been reported at the recipient's hand.^5^

However, the donor foot presents many problems due to the great amount of tissue sacrificed.^6^, ^7^ Postoperative complications are not uncommon at the donor site after toe‐to‐hand transfer, such as pain, wound infection, scarring, or callosity at the foot. What is more, the esthetic role of the foot should not be underestimated in patients’ daily lives. The unsatisfactory appearance of the donor foot is the main reason why some patients were reluctant to receive this surgery.

Therefore, how to minimize the amount of tissue sacrificed of the donor foot becomes an urgent problem to be addressed.^8^, ^9^ In Yamada's study, skin expansion is applied on the great toe, aiming to minimize the residual skin defect after the wraparound flap is harvested.^10^ However, only skin tissue was increased through their method.

As is well‐known, the Ilizarov technique is widely used in distraction osteogenesis.^11^ Besides new bone formation, soft tissues around the bone could also be regenerated *via* slow distraction.^12^, ^13^ In our study, we tried to utilize the Ilizarov technique to enlarge the donor's great toe before finger reconstruction. The aims of this study were (i) to evaluate the feasibility and effectiveness of regenerating tissues of the great toe through transverse distraction, (ii) to evaluate the influence of the distraction on the digital arteries and nerves of the great toe, and (iii) to introduce operative tips and complications of the distraction surgery.

## Patients and Methods

### Patient Characteristics

From September 2020 to December 2022, 23 patients (30 toes) underwent transverse distraction of the great toe to enlarge the donor site for toe‐to‐hand transfer. Patients were selected to undergo this surgery when they met the following criteria: (i) preparing to receive finger reconstruction with partial great toe transfer; (ii) there was high demand for function and appearance at both recipient and donor sites; (iii) understanding the potential complications of distraction osteogenesis. This study was approved by the ethical committee of our hospital (SWYX: No. 2021–487), and all participating patients provided written informed consent.

Study participants included 10 male and 13 female patients with an average age of 28.9 years (7–45 years old). Their defective fingers consisted of three thumbs, eight index fingers, seven middle fingers, seven ring fingers, and five little fingers. The widths of the contralateral normal fingers were measured, including the finger nail (14.1 ± 2.7 mm) and the distal phalanx (13.2 ± 2.3 mm), which were set as the objectives for the distraction enlargement of the donor great toes. All the surgeries were performed by the same senior surgeon (Dr. Liwen Hao).

### Operative Technique

**FIGURE 1:**
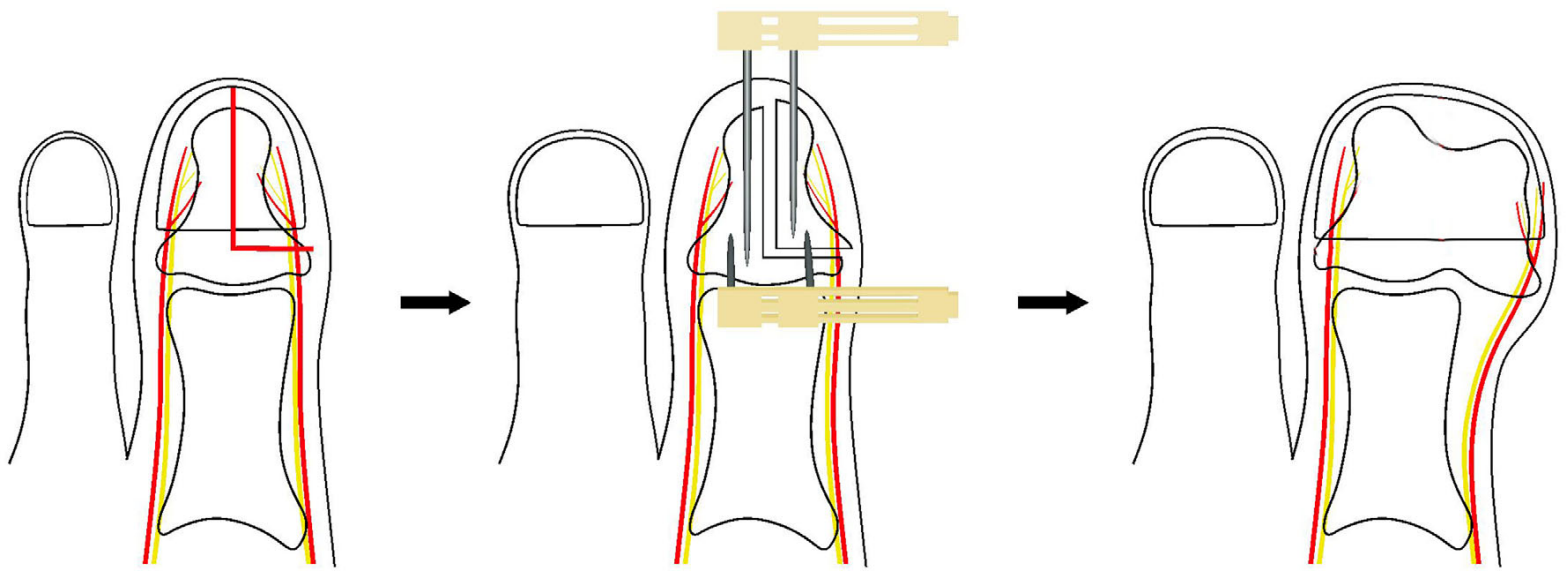
Surgical diagram.

**FIGURE 2:**
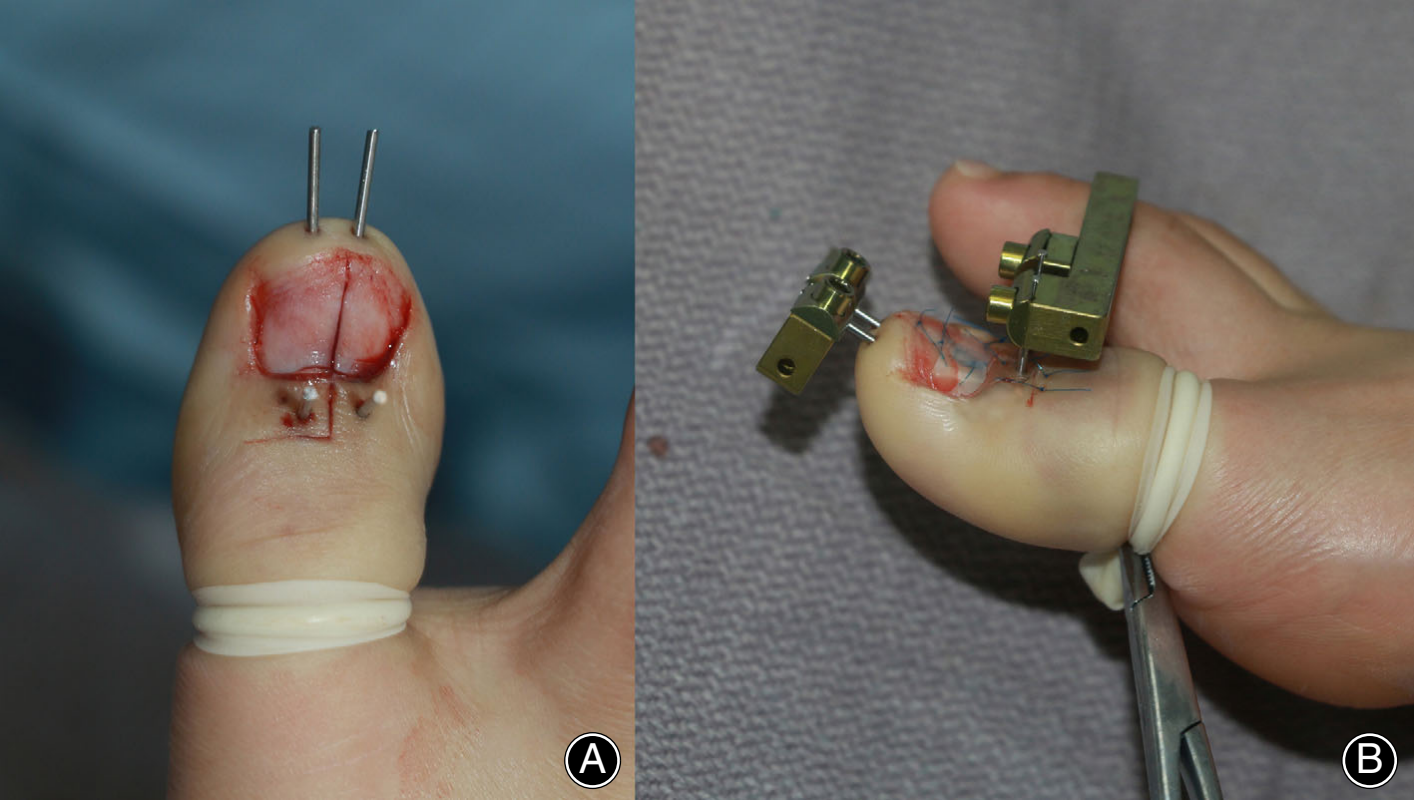
(A) L‐shaped incision and four Kirschner pins on the great toe; (B) two mini external fixation frames were installed on the pins.

The graphical abstract image of the operative technique is shown in Figure 1. After local anesthesia, the nail plate of the great toe was carefully removed. Then, an L‐shaped incision was made on the dorsal aspect of the distal great toe for the following osteotomy (Figure 2A). The longitudinal part of the incision is along the midline of the nail bed, and the transverse part is at the medial side of the midline, which is between the nail matrix and the interphalangeal (IP) joint.

**FIGURE 3:**
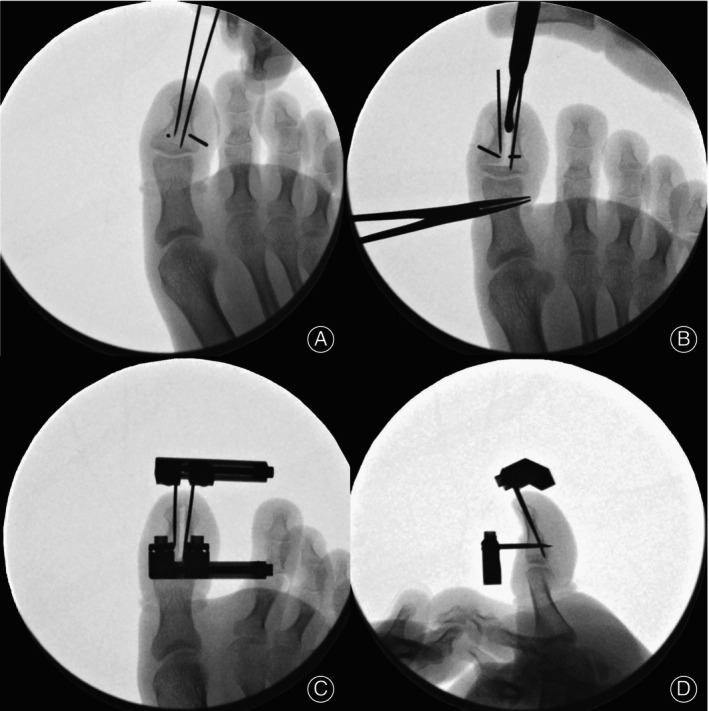
Intraoperative fluoroscopy. (A) four Kirschner pins were inserted on the distal phalanx; (B) osteotomy was performed on the distal phalanx; (C) dorsal view; and (D) lateral view of X‐ray after external fixation frames were installed.

Before osteotomy, four Kirschner pins (1.2 mm in diameter) were inserted onto the great toe in advance (Figure 2A). To be specific, two longitudinal pins were inserted parallel with a distance of about 5 mm in the distal phalanx. The tip of the lateral pin was close to the IP joint, and the medial one was shorter than the lateral one. Two perpendicular pins were inserted at the base of the distal phalanx, bilateral to the longitudinal ones. The tips of the perpendicular pins penetrated out of the plantar cortex of the phalanx slightly. Repeated intraoperative fluoroscopy helped precisely locate these pins on the distal phalanx (Figure 3A). Then, the Kirschner pins were shortened to facilitate osteotomy.

Along the L‐shaped incision, osteotomy was performed using an oscillating saw with a 5‐mm wide blade. The longitudinal cutting is along the midline of the distal phalanx, and the transverse cutting is about 5‐mm distal to the IP joint (Figure 3B). Then, two mini external fixation frames (Xinzhong Medical Instrument Co., Tianjin, China) were installed (Figure 2B). One frame was placed on the longitudinal pins at the tip of the toe, and the other one was placed on the perpendicular pins at the dorsal side of the great toe (Figure 3C,D). Transverse distraction was performed intraoperatively to confirm complete corticotomy. After the osteotomy site was adjusted to its original position, the nail bed and skin wounds were sutured.

### Postoperative Treatment

About 1 week after the surgery (latency period), patients were instructed to adjust the fixation devices by themselves (active distraction period). The suggested widening rate was 0.5 mm per day, and the frequency was twice daily with 0.25 mm each time. If patients cannot tolerate the pain caused by distraction, the rate was slowed down to 0.25 mm per day. The transverse distraction of the great toe was stopped when the objective width was achieved. The fixation devices were kept in place until complete osteogenesis was confirmed by X‐ray examination (consolidation period). During the treatment time with external fixation, patients were encouraged to walk with forefoot, offloading shoes.

### Observational Index

Before the surgery and after the distraction enlargement, the width of the phalangeal tuberosity of the great toe was measured on X‐ray images. The width of the toenail and plantar pulp of the great toe were measured with a Vernier caliper. Doppler ultrasound was used to assess the integrity of digital arteries and nerves of the great toe after distraction enlargement. Computed tomography angiography (CTA) was performed to further confirm the location of digital arteries of the great toe as a preparation for the following finger reconstruction.

Postoperative complications were recorded, including major ones that need revision surgery (such as pin loosening, premature or failed consolidation, and callus fracture) and minor ones that do not need surgery (such as pin‐track infection, wound healing problems, and joint stiffness).

### Statistical Analysis

The data were presented as the mean value and standard deviation. Paired *t*‐test was used to compare the measurements of the great toe before and after the surgery. Statistical analyses were carried out using Statistical Package for the Social Sciences (SPSS) software (SPSS 19.0, IBM, New York, NY, USA), and the level of statistical significance was set at *p* < 0.05.

## Results

### Time for Distraction

The time for active distraction is 46.1 ± 8.3 days (range, 35–72 days) with a widening rate of 0.41 ± 0.08 mm/day (range, 0.25–0.5 mm/day). The time for consolidation is 30.9 ± 5.5 days (range, 20–45 days), and all patients had successful osteogenesis. Counting in the latency period, the time for treatment with external fixation was 84 ± 11.9 days (range, 62–115 days).

### Change of the Great Toe after Distraction

**FIGURE 4:**
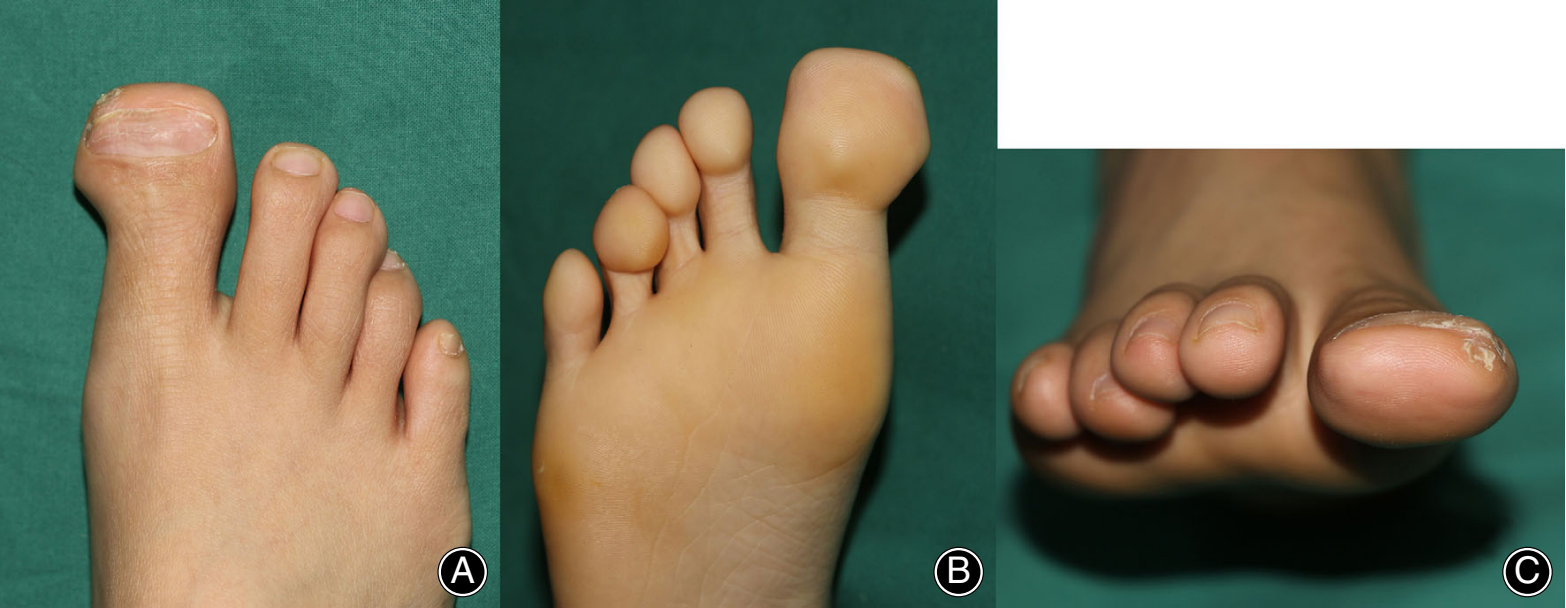
Appearance of the great toe after transverse enlargement. (A) Dorsal view; (B) plantar view; and (C) view from the toe tip.

**FIGURE 5:**
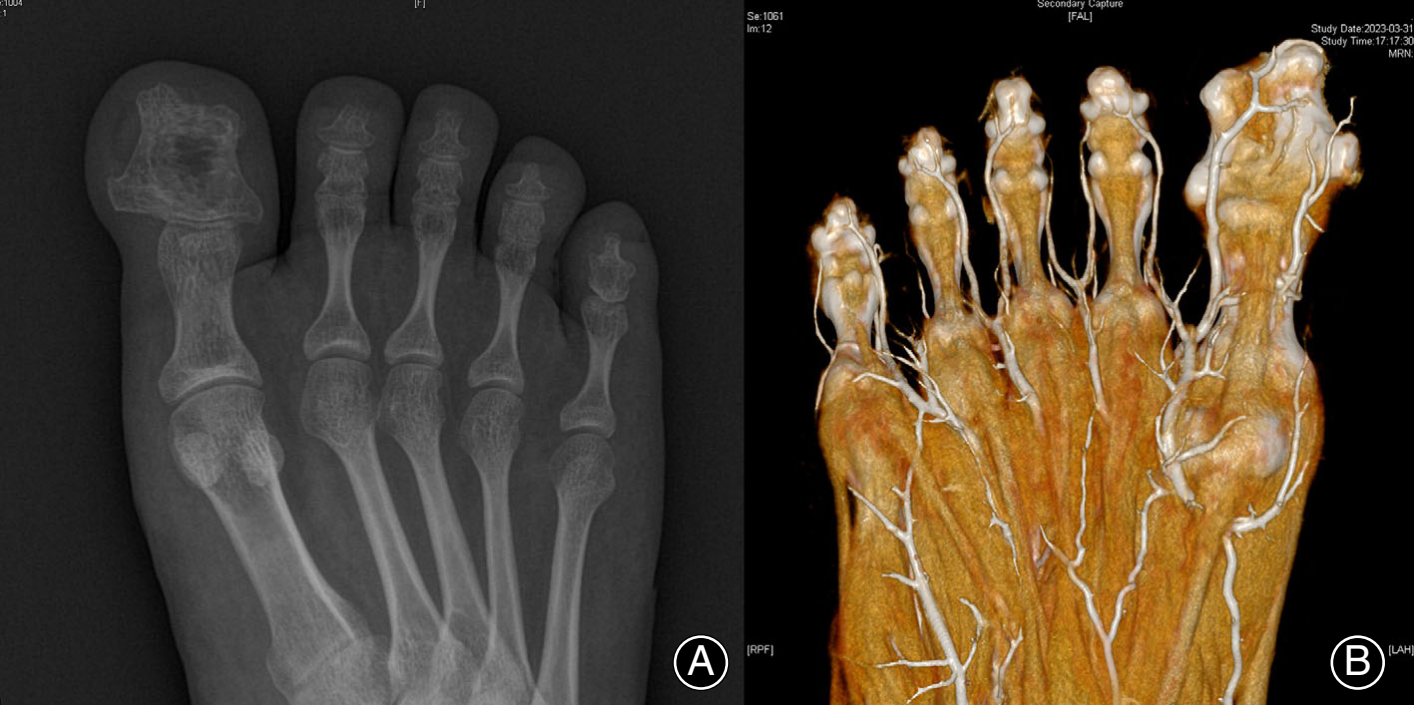
(A) X‐ray and (B) CTA of the great toe after transverse enlargement.

After the distraction enlargement, the width of the distal phalanx of the great toe increased from 13.1 ± 1.5 mm to 28.1 ± 2.8 mm (*p* < 0.001). The width of the toenail increased from 15.8 ± 1.7 to 30.3 ± 3.0 mm (*p* < 0.001), and the width of the plantar pulp increased from 25.6 ± 3.1 to 38.8 ± 3.2 mm (*p* < 0.001). Figures 4 and 5A show the appearance and X‐ray of the enlarged great toe after transverse distraction.

Doppler ultrasound showed good patency of both medial and lateral digital arteries in all the patients after distraction, and the digital nerves on both sides were intact. CTA (Figure 5B) showed that the medial digital artery moved with the bone fragment to the medial side, and the lateral digital artery stayed at its original position.

### Complications

Regarding major complications, one patient needed revision surgery due to pin loosening, and another one needed re‐osteotomy due to premature consolidation. The minor complication was mainly pin‐track infections, which were observed in 5 out of the 30 toes.

## Discussion

In finger reconstruction, minimizing the amount of tissue sacrificed at the donor site has always been challenging because composite osteo‐onychocutaneous tissues will be harvested from the donor foot.^14^, ^15^ In our patients, multiple tissues of the great toe, including bone, nail, and skin, were effectively regenerated after transverse distraction.

### Regenerating Multiple Tissues of the Great Toe

As is well known, the Ilizarov technique is widely used to stimulate bone regeneration. Numerous studies reported satisfactory results of bone lengthening through longitudinal distraction.^13^, ^16^ In our study, transverse distraction was performed on the distal phalanx, and similar distraction osteogenesis was obtained. In traditional partial great toe transfer, especially for thumb reconstruction, most of the distal phalanx is harvested, and only a narrow bone fragment is left at the donor site.^17^ Through our method, the distal phalanx could be widened until enough bone is regenerated. After toe‐to‐finger transfer, the almost normal width of the phalangeal bone at the great toe was preserved.

In finger and thumb reconstruction, the transferred nail is important in cosmetic appearance and refined function of the hand.^15^ However, at the donor site, most or all of the toenail will be lost. Some patients are reluctant to wear open‐toed shoes or even refuse to receive toe‐to‐hand transfer due to the unsatisfactory appearance of the donor foot. In our patients, the nail apparatus at the great toe, including the nail bed and matrix, was effectively widened after transverse distraction. Larger part of the toenails will be preserved after nail harvesting for finger reconstruction. It is worth noting that the new nail apparatus was regenerated with the underlying bone, and the nail will be transferred with the bone in the following surgery, which could prevent iatrogenic injury to the nail bed and matrix.

Stable and sensitive plantar skin of the foot is important for weight‐bearing and walking.^18^ Many postoperative complications after the toe‐to‐hand transfer, such as pain and callus formation, are related to the lack of normal weight‐bearing skin on the donor foot.^6^, ^9^ Therefore, the amount of plantar skin harvested from the toe is deliberately restricted. In our patients, the plantar pulp of the great toe was also effectively widened after transverse distraction. More weight‐bearing skin will be preserved after the flap is harvested, which will help decrease donor site complications. What is more, more skin left on the toe increasing the skin left also facilitates donor site coverage. Skin graft or dressing change will be enough for the residual defect instead of flap repair at the donor foot.

### Influence on the Digital Arteries and Nerves of the Great Toe

Our results have confirmed that the digital vessels and nerves of the great toe are undamaged after distraction surgery. However, previous studies have proved that tibial cortex transverse transport could improve local microcirculation through vascular tissue regeneration.^19^, ^20^ Therefore, it is reasonable to assume a positive effect of distraction osteogenesis on the blood supply of the great toe, but further studies are needed.

### Operative Tips and Complications of the Distraction Surgery

Several tips should be paid attention to during the distraction surgery. First, considering the limited space between the first and second toes, the medial side was chosen as the direction of transverse distraction. Second, pin insertion and osteotomy on the distal phalanx should be done with extreme caution, especially for female patients with small feet. A small saw blade and repeated X‐ray scanning were necessary. Third, two mini external frames were needed. When only one frame was installed at the toe tip, the distraction in the distal part was wider than that in the proximal part. To solve this problem, a proximal dorsal frame was added.

Similar to other Ilizarov techniques, there are some intrinsic shortcomings of distraction surgery, such as prolonged treatment and inconvenience with external fixation.^12^, ^13^ Postoperative complications, such as pin loosening and premature consolidation, were also found and need re‐surgery. Patients should be fully informed about the increased time and financial costs of treatment, and only well‐motivated patients were selected. Individuals who ask for good functional and esthetic results from both donor and recipient sites may benefit from this surgery.

### Strengths and Limitations

Through transverse distraction, composite osteo‐onychocutaneous tissues of the great toe were effectively regenerated without disturbing the digital arteries and nerves. To the best of our knowledge, this is a novel method to truly enlarge the donor toe before finger reconstruction. We are also aware that our study may have two limitations. The first is the inherent limitation of a retrospective study. The second is that the exact influence of distraction surgery on the following finger reconstruction is unclear, such as potential bone healing problems, arterial or venous insufficiency, and the satisfaction of appearance, which will be the focus of our future study.

## Conclusion

With the help of the Ilizarov technique, the great toe was effectively enlarged after transverse distraction. Multiple tissues of the great toe, including bone, nail, and plantar skin, were regenerated via distraction osteogenesis, and more tissues were preserved after the partial great toe flap was harvested. This procedure could help to minimize the amount of tissue sacrificed of the donor foot for toe‐to‐hand transfer. Further study on the influence of distraction enlargement on the following microsurgery is needed.

## Author Contributions

Liwen Hao, Kai Rong, Chao Chen, and Zhidian Hou: Collected the raw data and prepared the manuscript. Xu Tian: Performed statistical analysis and interpreted the data. Yaxing Wang: Performed radiograph analysis. Zengtao Wang: Designing the study. All authors have read and approved the final manuscript.

## Authorship Declaration

We declare that all authors listed meet the authorship criteria according to the latest guidelines of the International Committee of Medical Journal Editors. All authors agreed to the final submitted manuscript.

## Conflict of Interest Statement

All authors have no conflicts of interest in this work.

## Ethics statement

The study protocol was approved by the Ethics Committee of Shandong Provincial Hospital (SWYX: No. 2021–487). Written informed consents were obtained from all participants.
